# Body-Fat-Percentile Curves for Thai Children and Adolescents

**DOI:** 10.3390/nu15020448

**Published:** 2023-01-14

**Authors:** Maneerat Puwanant, Ladda Mo-Suwan, Somchit Jaruratanasirikul, Wipa Jessadapakorn

**Affiliations:** 1 Department of Pediatrics, Faculty of Medicine, Prince of Songkla University, Hat Yai District, Songkhla 90110, Thailand; 2 Vachira Phuket Hospital, 353 Yaowarat Road, Talat Yai Subdistrict, Mueang Phuket District, Phuket 83000, Thailand

**Keywords:** body fat, reference curves, children, adolescents, obesity

## Abstract

This study aimed to generate sex-specific percentile curves for the percentage of body fat (PBF) in Thai children using a bioelectrical impedance analysis (BIA). The secondary objective of this study was to determine the association between body fat and other anthropometric measurements. A cross-sectional study was conducted on 3455 Thai school children aged 6–18 years. The body-fat contents were measured using BIA. Smoothed percentile curves of PBF were derived using a scatter plot with a smooth curve fitted by the Loess method. The correlations between the body fat and the anthropometric measurements were assessed using the Spearman’s rank correlation. The 50th and lower body-fat-percentile curves of the boys slowly increased until age 12, after which they slightly decreased until age 15 and then slightly increased until age 18. In the higher boys’ percentiles, the body fat sharply increased until age 11 and then decreased until age 18. In the girls, the PBF percentiles increased steadily from 6 to 18 years. The body-mass index was strongly correlated with PBF and fat mass in both sexes. The waist-to-height ratios showed strong correlations with PBF and fat mass in the boys but were moderate in the girls. The use of PBF percentile curves can be an additional metric for the evaluation of obesity in Thai children.

## 1. Introduction

Childhood obesity is widely considered a serious global health problem and its worldwide prevalence has increased markedly over the past decade [1]. Similarly, the prevalence of childhood overweight and obesity in Thailand rose sharply from 5.8% in 1995 in 6–12-year-old children to 13.9% in 2014 (almost triple that in 1995) in 6–14-year-old children [2].

Obesity is a state of excessive accumulation of body fat [3], the assessment of which is an important step in developing preventative and treatment programs for childhood obesity. The body-mass index (BMI) is widely used to assess obesity status as it correlates well with adiposity, as measured by dual-energy X-ray absorptiometry (DXA), in both children and adults [4,5,6]. However, BMI does not directly measure body fat and does not distinguish weight associated with lean tissue and bone from body fat, which can lead to the misclassification of overweight and obesity [6]. Among the various methods used to measure body fat directly, underwater weighing and DXA are the standard methods; however, they are complex, expensive and time consuming and, thus, impractical in a clinical setting. Skinfold-thickness measurement is a simple and noninvasive method for estimating subcutaneous body fat, but technician expertise is required for accurate assessment.

In recent years, bioelectrical impedance analysis (BIA) has become a popular method for measuring body composition. It is simple to use and requires no specialized facilities or expertise to be conducted. It can also be performed in a clinical setting or in field surveys. Additionally, it can be performed quickly and easily and is less expensive than underwater weighing or DXA [7]. Bioelectrical impedance analysis can distinguish between lean and fat tissue based on the differences in electrical conductivity within fat mass and fat-free mass [7,8]. An electrical current passes quickly through fat-free mass because it has a high water content, but meets resistance when flowing through fat mass because it has a lower water content. This resistance to electrical current, known as impedance, is measured and input into specific equations according to age, sex, weight, height and electrical impedances to calculate body composition. One study reported that the percentage of body fat (PBF) in children measured by BIA was significantly correlated with underwater weighing, which is regarded as the gold standard for measuring body composition (*r* of 0.79 and 0.69 in girls and boys, respectively; *p* ≤ 0.010) [9]. Therefore, fat mass, as measured by BIA, denoted as PBF, is widely used to assess health risks from obesity in children.

Differences in body composition may exist in different ethnic groups owing to the effects of environmental and genetic differences that are specific to different populations [10]. Body-fat reference curves have been constructed for many countries; however, data on Thai reference values for body fat in children are scarce and body-fat reference curves for Thai children have never been constructed. Therefore, the present study was carried out with the primary aim of generating sex-specific percentile curves for PBF in Thai children aged 6–18 years old using BIA analysis to assess children’s adiposity. The secondary objective was to determine the association between body fat and BMI and between body fat and the waist-to-height ratio (WHtR).

## 2. Materials and Methods

### 2.1. Participants and Study Design

**Figure 1:**
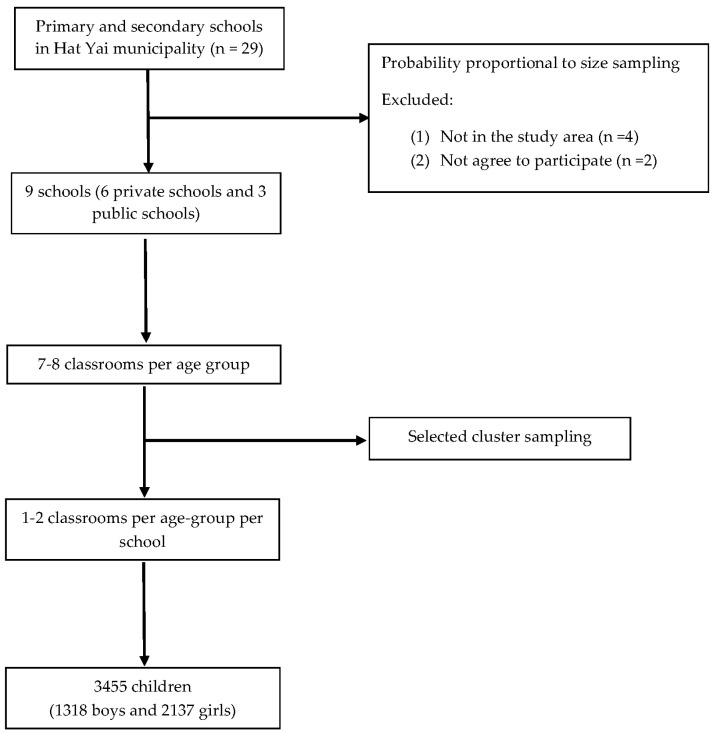
Flow chart of the study process, wherein participants were randomly selected by probability-proportional-to-size sampling.

A cross-sectional study was conducted from June 2011 to March 2012 in nine primary and secondary schools (six private schools and three public schools) in the Hat Yai municipality of southern Thailand, which were randomly selected by probability-proportional-to-size sampling from the 29 schools in this area. One to two classrooms in each age group were randomly selected using cluster sampling of classrooms from the school (Figure 1). A total of 3455 Thai schoolchildren (1318 boys, 2137 girls) aged 6–18 years were recruited for the study following contact with the school by the researchers. Children and adolescents with weights and heights between the 2.5th and 97.5th percentiles were selected. Those whose weight and height were lower than the 2.5th percentile or higher than the 97.5th percentile were removed as outliers to ensure homogeneous data distribution. Assent and informed consent were obtained from all the children and their parents. The study protocol was approved by the Institutional Review Board and the Ethics Committee of the Faculty of Medicine, Prince of Songkla University (EC 54-069-01-1-3-2).

### 2.2. Measurements

The measurements were performed by well-trained staff. Height was measured in the standing position without socks, shoes, or head accessories to the nearest 0.1 cm with a portable standard stadiometer. Weight and body composition were measured using a foot-to-foot body-composition analyzer, the Tanita SC-330 (Tanita, Corporation, Tokyo, Japan), using tetrapolar bioelectrical impedance analysis, with precisions of 0.2 kg and 2%, respectively. This analyzer measures body composition using a constant current source with a high-frequency current (50 kHz, 90 µA) and can measure PBF and fat mass to the nearest 0.1% and 0.1 kg, respectively. The children were asked to wear only their school uniforms without belts, socks, or shoes during the measurements. They stood barefoot on the analyzer. The BMI was calculated by dividing the weight (kg) by the square of the height in meters (kg/m^2^). Waist circumference (WC) was measured midway between the lowest rib and superior border of the iliac crest using a non-elastic measuring tape (Butterfly brand, Shanghai, China) to the nearest 0.1 cm. All data and measurements were collected by the same team and all instruments were calibrated daily.

### 2.3. Definition

Childhood obesity was diagnosed if BMI-for-age was greater than two standard deviations above the World Health Organization (WHO) Growth Reference 2007 median. Thinness was diagnosed if the BMI-for-age was less than two standard deviations below the WHO Growth Reference 2007 median. Stunting was diagnosed if height-for-age was less than two standard deviations below the WHO Growth Reference 2007 median [11].

### 2.4. Statistical Analysis

The participants were categorized by age at the midpoints of 6-month intervals (e.g., 7.5 years included children aged 7.25–7.74 years and 8 years included children aged 7.75–8.24 years). All statistical analyses were performed using R software v4.0.3 (R Foundation for Statistical Computing, Vienna, Austria, available online: https://www.R-project.org/ (accessed on 14 January 2021)). Continuous variables were expressed as mean and standard deviation and were compared between the sexes using Student’s t-test. Statistical significance was set at *p* < 0.05. Smoothed sex-specific percentile curves of PBF were created using a scatter plot with a smooth curve fitted using the Loess method. Correlations between PBF and fat mass by BIA and the outcome measures of BMI and waist-to-height ratio were assessed using Spearman’s rank correlation.

## 3. Results

### 3.1. Overall Sample Characteristics

A total of 3455 schoolchildren (38% boys and 62% girls) aged 6–18 years were included in this study. The overall prevalence of obesity based on the WHO Growth Reference 2007 was 12.6% (19.7% in boys and 8.3% in girls): it was 17.1% in children under 10 years old, 18.9% among 10–12-year-olds, 10.2% among 13–15-year-olds and 6.2% in children over 15 years old (Table 1). The overall prevalence of thinness and stunting was 5.0% and 4.3%, respectively.

The age- and sex-specific mean anthropometric parameters, including weight, height, BMI, WC and PBF, are shown in Table 2. There were no differences in BMI between the boys and the girls in most of the age groups, with the exception of the 7-, 10- and 11-year groups, in which the boys had higher BMIs than the girls. The body-mass index increased with age, from around 16 kg/m^2^ in the boys and 15 kg/m^2^ in the girls at age 6 to 21 kg/m^2^ at age 15 in both sexes, after which it remained fairly stable until 18 years of age. The boys had greater waist circumferences and waist-to-height ratios than the girls in most of the age groups. The percentage of body fat, as measured by BIA, was significantly greater in the girls aged between 12 and 18 years. Similarly, the girls had significantly larger fat mass at ages 10, 12 and 14–18.

### 3.2. Percentage of Body Fat in Percentiles and Percentile Curves for PBF

The P_50_ of the PBF among the boys increased from ages 6 to 10 years and subsequently tended to decrease until 18 years of age, whereas the P_50_ of the PBF among the girls generally increased from ages 6 to 18 years. The full set of PBF percentiles (P_3_, P_10_, P_25_, P_50_, P_75_, P_85_, P_90_, P_95_ and P_97_) according to age and sex is shown in Table 3.

**Figure 2:**
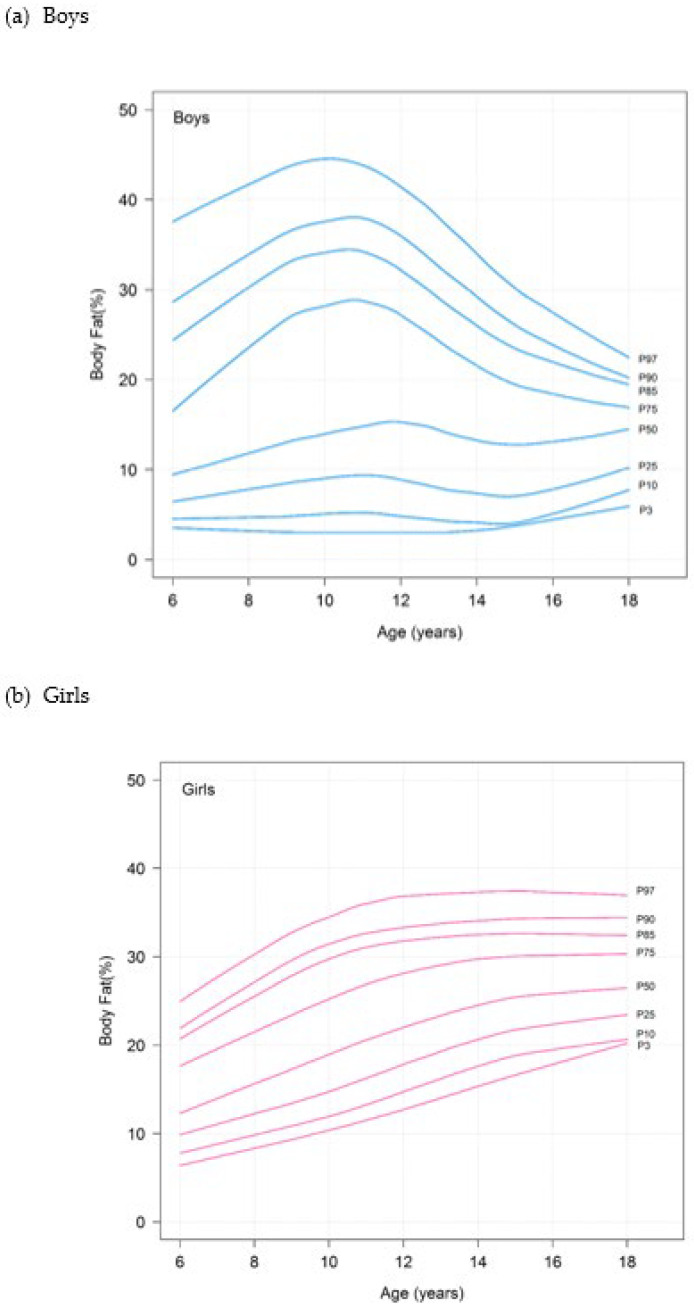
Percentile curves for percentage body fat of (**a**) boys and (**b**) girls.

Smoothed age- and sex-specific percentile curves for PBF are shown in Figure 2. The shapes of the PBF percentile curves differed significantly by sex. In the boys, the third percentile was fairly stable from ages 6 to 14 years and then slightly increased until the age of 18 years. The 50th percentile and the remaining lower percentiles of the boys slightly increased until age 12, slightly decreased from ages 12 to 15 years and subsequently repeatedly increased slightly until the age of 18 years. However, the higher percentiles of the boys sharply increased until age 11 and then decreased until age 18. In the girls, the percentages of the body-fat percentiles increased steadily from 6 to 18 years, with the exception of the 85th, 90th and 97th percentiles, which increased until 12 years of age and then remained fairly stable from 12 to 18 years of age. The median PBF percentile of the boys was lower than that of the girls.

### 3.3. Correlations between Body Fat and Anthropometric Measures

Table 4 shows the Spearman’s correlation coefficients used to determine the correlations between body fat and anthropometric variables, that is, BMI and waist-to-height ratio. Body-mass index was strongly correlated with PBF and fat mass in both the boys (*r =* 0.82 and 0.95, respectively) and the girls (*r =* 0.97 and 0.97, respectively). The waist-to-height ratio was strongly correlated with PBF and fat mass in the boys (*r =* 0.89 and 0.81, respectively), but these correlations were moderate in the girls (*r =* 0.78 and 0.66, respectively).

## 4. Discussion

The present study is the first to report age- and sex-specific percentile curves for PBF measured by BIA in Thai children and adolescents between 6 and 18 years of age using a large sample from Southern Thailand. We found that the percentile curves for the PBF showed significant sex differences; the girls appeared to have increased values with age, while the values in the boys tended to decrease after 10–11 years of age. These sex differences in body fat normally occur in all human populations. They are physiologically attributed to the effects of hormones during puberty, during which estrogen and progesterone increase the number of adipocytes in females, whereas androgens lead to a decrease in fat mass in males during adolescence. In general, males attain a peak percentage of fat during early puberty, which then decreases during adolescent growth, contrary to females, who show an initial decline in fat levels, followed by a continuous increase in fat percentage thereafter [12]. According to Jaruratanasirikul, et al. [13], the age of onset of puberty in Hat Yai schoolboys (the same study population as that in the present study), was 10.6 years, which corresponded to the peak mean PBF in the boys that occurred at 10 years of age and subsequently gradually decreased in this study. Similarly, the median ages of Hat Yai schoolgirls with thelarche and menarche in another study were 9.6 and 12.2 years [14], respectively, which conformed with the pattern of PBF in the girls as their adolescence progressed in our study.

**Figure 3:**
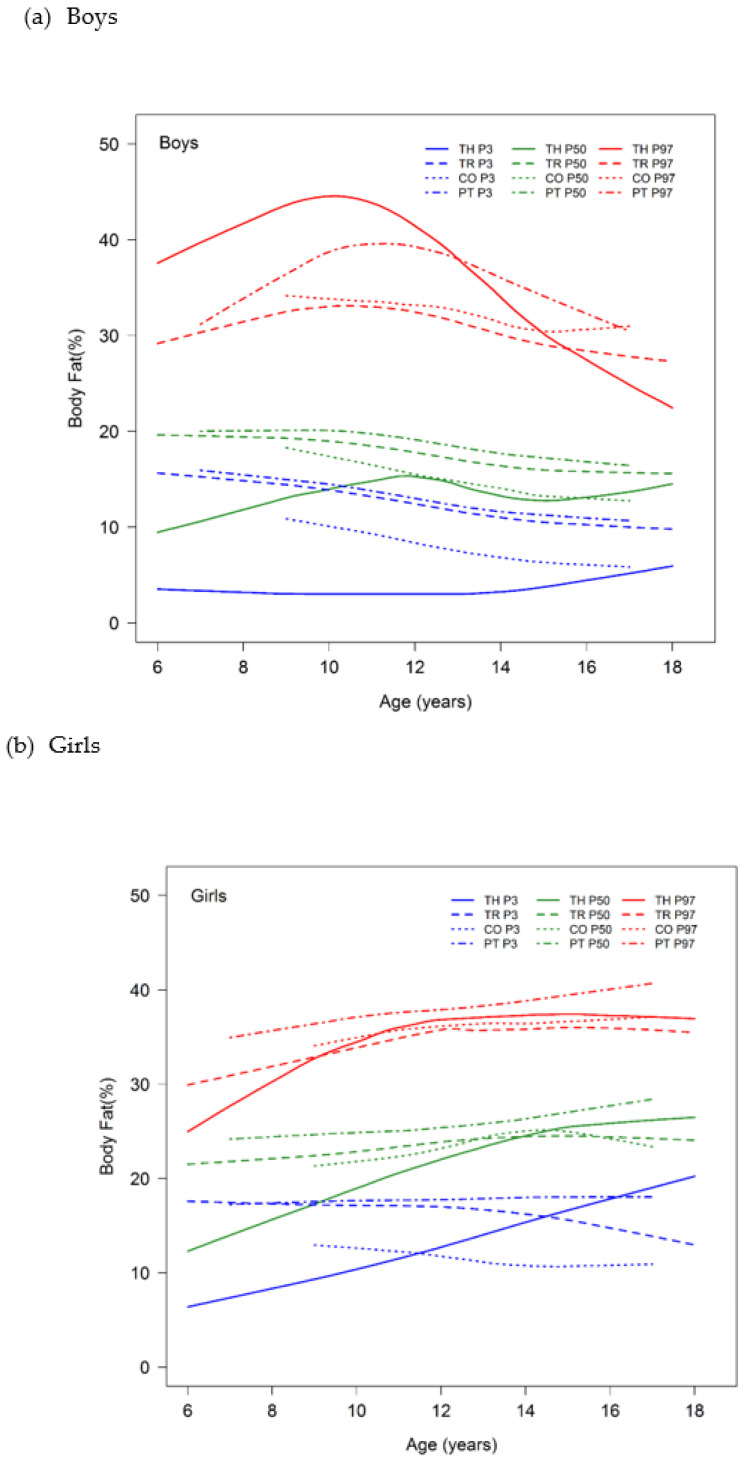
The 3rd, 50th and 97th percentiles for percentage body fat according to age in Thai, Turkish, Colombian and Portuguese (**a**) boys and (**b**) girls. Abbreviations: TH, Thai; TR, Turkish; CO, Colombian; PT, Portuguese.

The 3rd-, 50th- and 97th-percentile curves for PBF in our study were compared with percentile curves from studies conducted in Turkey, Colombia and Portugal that used the same BIA instrument device [10,15,16] (Figure 3). Among boys, the 50th- and 3rd-percentile curves of the Thai children were significantly lower than those of Turkish, Colombian and Portuguese boys at 6 years of age, while the 50th- and 3rd-percentile curves of Thai boys became close to those of other populations at 12–18 years old and 17–18 years old, respectively. However, the 97th-percentile curve was significantly higher than those observed in children from Turkey, Colombia and Portugal at 6 years of age, increased to a peak at 10 years of age and then progressively decreased, becoming similar to the corresponding Portugal curve for 13-year-olds and the Colombia and Turkey curves for 15-year olds. The patterns of the 50th- and 3rd-percentile curves of the Thai boys were quite different from those of the other countries. The 50th-percentile curve of the Thai boys progressively increased from 6 to 12 years and then decreased until 15 years and slightly increased again from 15 to 18 years of age, while this curve in the other populations continually decreased throughout all ages. The 3rd percentile of Thai boys was stable until 14 years and then slightly increased from 14 to 18 years of age, while this curve also decreased throughout all ages in the other populations. The pattern of the 97th percentile of Thai boys was close to that of the Turkish and Portuguese boys, which was a standard bell curve. The difference between the values and patterns of the PBF curves of the Thai boys and those representing other populations from different continents may be due to ethnic differences. The pattern of the PBF curves of the Thai boys was different not only from the curves of other countries in our comparisons but also from those of other South and East Asian countries, namely, India and Hong Kong, respectively. Sung et al. reported that the PBF curves in Hong Kong boys were fairly stable from ages 6 to 18 [17], while Chiplonkar et al. found that the 50th-percentile curves in Indian boys increased from 5 to 12 years of age, after which they declined up to 17 years of age [18]. This pattern was close to that of the PBF curves of our Thai boys, except that the percentile curves of the Thai boys declined from 12 to 15 years and then slightly increased from 16 to 18 years of age.

In our Thai girls, the 50th- and 97th-percentile curves were similar to those of the countries noted above and increased with age [10,15,16]. Both percentile curves of the Thai girls were significantly lower than those of the Turkish, Colombian and Portuguese girls at 6 years of age but progressively increased and overlapped with the corresponding the 50th-percentile curves for the Turkish and Colombian 14- and 15-year-olds, as well as with the 95th-percentile curves for the 9- and 10-year-olds, respectively; they were then slightly higher than the Turkey and Colombia curves after these ages. The 50th- and 97th-percentile curves of the Thai girls were significantly lower than those of the Portuguese girls at 6 years of age but progressively increased and later became slightly lower than those of the Portuguese adolescents at 14 and 12 years of age, respectively. The 3rd-percentile curves of PBF in the Thai girls increased for all ages; however, the 3rd-percentile curves for Turkey, Colombia and Portugal were fairly stable or slightly decreased for all ages. When we compared the 3rd-percentile curves in the female children, the curve of the Thai girls was significantly lower than that of the Turkish, Colombian and Portuguese girls at 6 years of age but progressively increased and overlapped the corresponding Colombian, Turkish and Portuguese curves at 11.5, 14.5 and 16 years of age, respectively, after which it became higher than these curves. Additionally, the patterns of all the percentile curves of PBF of the Thai girls were close to those of the Hong Kong and Indian girls, which are Asian populations, although these Asian curves were assessed using other brands of BIA instruments [17,18].

These differences in PBF between Thai children and adolescents and those from other populations may be due to ethnic differences in fat accumulation during growth [12]. Therefore, ethnicity-specific references for body composition are needed to evaluate the body fat in each country. In addition, these differences may be related to the different BIA methods used to estimate the PBF. Although our comparison of the percentile curves with those of other countries used the same brand of BIA machines, the studies all used different methods of assessment. For example, the present study and the study from Colombia used foot-to-foot measurements, whereas the studies from Turkey and Portugal used hand-to-foot measurements [10,15,16]. Moreover, the prevalence of obesity and thinness was different in each country, which might have affected the PBF values. Finally, there are environmental and cultural differences in every country, including nutritional trends and physical-activity levels, which can affect anthropometric measurements.

The body-mass index showed strong correlations with PBF and fat mass, as measured by BIA, in both sexes. The waist-to-height ratios showed strong correlations with PBF and fat mass in the boys but moderate correlations in the girls. These results are consistent with those from many other studies that have evaluated the associations between anthropometric variables, that is, BMI and waist-to-height ratios and adiposity in children [19,20,21,22]. Ramírez-Vélez et al. assessed the correlations between PBF using BIA (Tanita BF-689^®^ and Tanita BC-418^®^) and BMI in 1165 children and adolescents from Bogotá (Colombia) and found that BMI was significantly correlated with both PBF–BIA measures in both sexes, particularly in the 9–11-years group, in which BMI was strongly correlated with PBF (correlation coefficients = 0.814 and 0.852 in boys, 0.915 and 0.928 in girls for Tanita BF-689^®^ and Tanita BC-418^®^, respectively) [21]. Costa-Urrutia et al. also found that the relationship between PBF and BMI was strong in schoolchildren and adolescents (all cases R^2^ > 0.70) but not in preschool children (girls R^2^ = 0.57, boys R^2^ = 0.23) [22]. Jansen et al. found that BMI was strongly correlated with body fat calculated by BIA and that there was a moderate positive correlation with PBF, as calculated by DEXA, air-displacement plethysmography and isotope dilution [19]. Martin-Calvo et al. found that both BMI and waist-to-height ratio were strongly correlated with body fat measured using DEXA, which is the gold standard for assessing body composition in children [20]. Although BMI and waist-to-height ratio may not be ideal methods for evaluating body fat, as they cannot differentiate between fat mass and fat-free mass, both may be useful to define obesity when more sophisticated techniques, such as DEXA and BIA, are not available. 

This is the first study to provide body-fat percentiles measured by BIA in Thai children and adolescents aged 6–18 years of age. The strength of this study is that the sample size was large and the participants were randomly selected using a well-prepared sampling method to minimize selection bias. Furthermore, the anthropometric and body-composition measurements were highly reliable due to the use of a standard method. 

This study had some limitations. First, it was a cross-sectional study that was conducted in only one city in southern Thailand and, thus, might not represent all Thai children and adolescents. The second concern might be that this study did not include other potential outcome measurements, such as socioeconomic level, diet history and physical activity level, which may affect body composition. Lastly, since there were no national reference values for BMI in Thai children at the time of the study, the diagnoses of obesity, thinness and stunting in the present study were assessed by using BMI values based on WHO references, which may not accurately represent the nutritional status of Thai children and adolescents. 

The clinical implications of the study are worth mentioning. The determined PBF values can provide additional and useful information to evaluate childhood obesity in Thailand and can also be used to help predict cardiometabolic risks because they measure body fat directly. The percentile curves for PBF in our study can be applied as reference data for comparisons with other children in other regions of Thailand and will also be of use to pediatricians and others involved in assessing body fat in children, particularly obese children. Although there is no consensus on the diagnosis of obesity using PBF [16], the 85th percentile of PBF has been suggested as a cut-off for the definition of excessive body fat [17,23]. Therefore, PBF measured by BIA may be a reliable alternative method for evaluating childhood obesity in clinical practice.

## 5. Conclusions

The present study provided PBF percentile curves for Thai children and adolescents using BIA, which showed significant differences between the sexes. The PBF percentile curves from this study can be used as additional information for the clinical evaluation of obesity in Thai children and adolescents. Additionally, we found that the BMI and waist-to-height ratio were moderately-to-strongly correlated with PBF and fat mass, which may be useful in helping to define obesity when more sophisticated techniques are not available. Further studies from other parts of Thailand are required to develop a national reference set for Thai children and adolescents.

## Figures and Tables

**Table 1:** Prevalence of obesity in study children by sex and age group.

Age (Years)	Total			Boys			Girls		
	N	Obese	%	N	Obese	%	N	Obese	%
<10	895	153	17.1	391	96	24.6	504	57	11.3
10–12	694	131	18.9	313	84	26.8	381	47	12.3
13–15	911	93	10.2	401	63	15.7	510	30	5.9
>15	955	59	6.2	213	16	7.5	742	43	5.8
Total	3455	436	12.6	1318	259	19.7	2137	177	8.3

**Table 2:** Mean (standard deviation) anthropometric measurements and percentage body fat, according to age and sex, in the boys and girls.

Age (Years)	N		Weight (kg)			Height (cm)			Body-Mass Index (kg/m^2^)			Waist Circumference (cm)			Waist-to-Height Ratio			Percentage Body Fat (%)			Fat Mass (kg)		
	Boys	Girls	Boys	Girls	*p*-Value	Boys	Girls	*p*-Value	Boys	Girls	*p*-Value	Boys	Girls	*p*-Value	Boys	Girls	*p*-Value	Boys	Girls	*p*-Value	Boys	Girls	*p*-Value
6	55	41	22.67 (5.92)	20.25 (4.18)	0.028	117.16 (5.36)	114.84 (4.8)	0.031	16.38 (3.47)	15.18 (2.25)	0.056	55.79 (8.65)	52.48 (6.07)	0.039	0.48 (0.07)	0.46 (0.04)	0.108	14.29 (11.88)	13.9 (6.6)	0.854	3.88 (4.66)	3.05 (2.08)	0.291
7	121	135	24.76 (6.58)	22.57 (5.65)	0.005	121.35 (5.59)	119.44 (5.41)	0.006	16.63 (3.37)	15.68 (2.87)	0.016	57.71 (9.09)	54.29 (7.07)	<0.001	0.47 (0.06)	0.45 (0.05)	0.004	15.52 (11.31)	15.4 (7.21)	0.923	4.53 (4.44)	3.85 (3.12)	0.154
8	91	161	27.51 (9.17)	26.37 (6.54)	0.253	125.01 (5.62)	125.57 (5.51)	0.442	17.35 (4.47)	16.58 (3.15)	0.109	59.82 (10.56)	57.76 (8.31)	0.088	0.48 (0.07)	0.46 (0.06)	0.028	16.81 (13.97)	17.66 (8.06)	0.537	5.82 (7.67)	5.14 (3.89)	0.352
9	124	167	32.12 (9.7)	30.8 (8.47)	0.215	130.9 (5.98)	131.23 (6.82)	0.665	18.56 (4.67)	17.67 (3.64)	0.071	64.13 (12.16)	61.57 (9.55)	0.045	0.49 (0.08)	0.47 (0.06)	0.018	20.65 (15.56)	20.31 (8.93)	0.816	8.15 (8.28)	6.91 (5.01)	0.114
10	115	139	37.11 (9.46)	33.88 (8.71)	0.005	136.79 (6.22)	136.64 (6.82)	0.858	19.67 (3.96)	18.13 (3.69)	0.002	69.12 (10.68)	63.56 (9.66)	<0.001	0.5 (0.07)	0.46 (0.06)	<0.001	23.61 (12.86)	21.35 (8.86)	0.101	9.86 (7.45)	7.95 (5.36)	0.019
11	118	135	40.26 (11.83)	38.4 (10.66)	0.188	141.71 (6.87)	143.26 (7.51)	0.09	19.78 (4.62)	18.51 (4.01)	0.02	70.12 (12.48)	64.83 (10.17)	<0.001	0.49 (0.08)	0.45 (0.06)	<0.001	22.76 (14.23)	21.39 (8.86)	0.353	10.69 (9.1)	0.05 (6.83)	0.104
12	80	107	40.96 (11.61)	44.22 (11.97)	0.064	145.97 (8.43)	149.07 (6.03)	0.004	18.98 (4.17)	19.65 (4.44)	0.297	69.05 (12.04)	68.66 (11.25)	0.821	0.47 (0.07)	0.46 (0.07)	0.219	18.8 (12.57)	23.66 (9.25)	0.003	8.97 (8.3)	11.45 (8)	0.041
13	89	138	51.46 (14.41)	47.69 (10.34)	0.023	156.53 (8.4)	154.64 (6.52)	0.058	20.73 (4.38)	19.97 (3.57)	0.15	73.72 (11.77)	69.49 (8.86)	0.002	0.47 (0.07)	0.45 (0.05)	0.008	19.96 (11.46)	25.08 (7.46)	<0.001	11.66 (9.84)	12.69 (6.18)	0.33
14	129	159	54.08 (14.4)	49.66 (10.74)	0.003	162.32 (7.9)	155.15 (5.81)	<0.001	20.38 (4.5)	20.56 (3.88)	0.712	72.79 (12.38)	71.53 (9.59)	0.332	0.45 (0.07)	0.46 (0.06)	0.086	12.48 (10.79)	25.65 (6.99)	<0.001	7.88 (8.6)	13.42 (6.74)	<0.001
15	183	213	58.22 (14.79)	52.17 (10.91)	<0.001	165.3 (6.52)	156.95 (5.47)	<0.001	21.25 (4.88)	21.15 (4.09)	0.83	75.27 (12.6)	72.77 (9.12)	0.023	0.46 (0.07)	0.46 (0.06)	0.188	12.9 (8.46)	26.84 (6.92)	<0.001	8.67 (7.75)	14.72 (7.25)	<0.001
16	92	265	59.97 (13.31)	51.57 (10.24)	<0.001	168.29 (5.9)	156.8 (5.02)	<0.001	21.13 (4.26)	20.93 (3.66)	0.671	75.07 (10.23)	72.36 (8.44)	0.014	0.45 (0.06)	0.46 (0.05)	0.018	12.75 (7.24)	26.34 (6.16)	<0.001	8.46 (6.66)	14.2 (6.54)	<0.001
17	75	333	62.19 (12.77)	54.26 (12.5)	<0.001	169.54 (6.07)	157.3 (5.15)	<0.001	21.58 (3.93)	21.89 (4.72)	0.601	76.99 (10.52)	74.08 (10.54)	0.031	0.45 (0.06)	0.47 (0.07)	0.041	14.22 (6.53)	28.06 (7.49)	<0.001	9.59 (6.27)	16.09 (8.62)	<0.001
18	46	144	61.25 (13.08)	51.95 (9.44)	<0.001	169.05 (6.17)	157.68 (5.19)	<0.001	21.37 (4.04)	20.88 (3.51)	0.427	76.9 (10.47)	71.43 (7.98)	<0.001	0.45 (0.06)	0.45 (0.05)	0.892	15.05 (6.5)	26.49 (6.17)	<0.001	10.01 (6.85)	14.27 (6.11)	<0.001

**Table 3:** Percentage body fat in percentiles according to age and sex in the boys and girls in this study.

Age (Years)	N	Percentage Body Fat (%)								
		P_3_	P_10_	P_25_	P_50_	P_75_	P_85_	P_90_	P_95_	P_97_
Boys										
6	51	4.0	5.4	7.3	9.4	15.8	23.3	27.4	34.6	37.6
7	118	3.0	3.8	6.5	11.2	21.5	28.9	32.4	36.1	38.0
8	88	3.0	4.5	7.3	11.3	20.8	28.0	33.5	39.3	42.8
9	120	3.0	4.2	8.1	12.4	30.9	38.2	42.9	45.7	46.9
10	112	3.0	5.8	12.5	22.9	31.9	36.2	39.1	42.7	44.2
11	115	3.0	5.2	10.6	19.4	32.9	38.2	41.0	44.7	46.8
12	78	3.0	5.7	8.5	14.8	26.8	31.6	35.8	41.4	44.5
13	83	7.0	8.7	11.8	16.8	25.7	31.9	36.3	37.8	39.6
14	125	3.0	3.0	4.1	8.5	16.0	21.2	22.7	29.6	32.3
15	178	3.0	3.0	5.6	10.6	18.1	22.4	24.3	27.1	28.0
16	89	3.0	4.1	6.7	11.5	16.7	20.2	22.2	23.8	25.2
17	71	5.4	6.2	9.2	14.3	17.8	21.2	21.9	24.1	25.5
18	42	5.9	8.6	10.9	15.4	18.2	20.5	21.7	22.1	23.8
Girls										
6	38	6.4	7.7	10.5	12.8	17.8	21.1	21.7	23.5	23.8
7	127	7.1	8.9	10.5	13.3	19.3	21.4	23.4	26.9	28.1
8	151	8.7	10.0	11.8	15.1	21.6	26.1	28.2	30.4	30.9
9	157	9.4	10.8	13.4	18.5	25.5	29.4	31.3	35.0	35.4
10	131	10.0	12.0	15.7	19.3	26.0	31.3	33.1	35.5	37.4
11	128	11.1	12.7	15.4	20.0	25.4	30.1	31.7	33.9	36.9
12	101	12.6	13.9	17.0	21.9	29.0	33.0	34.1	36.0	36.8
13	130	14.3	16.7	20.1	25.4	29.9	32.5	33.7	35.7	36.4
14	151	15.4	18.6	21.4	24.6	29.6	32.0	34.1	36.6	37.8
15	202	16.6	19.2	22.5	26.5	30.4	33.6	35.2	36.9	38.0
16	251	17.9	19.6	22.1	25.7	29.9	31.3	32.6	35.6	37.4
17	315	19.0	20.8	23.3	26.7	30.8	34.5	37.8	40.7	42.2
18	136	17.4	19.7	23.1	25.9	29.9	31.6	33.8	35.9	36.7

**Table 4:** Correlations between body-mass index, waist-to-height ratio, percentage body fat and fat mass using Spearman’s rank correlation.

	Percentage Body Fat		Fat Mass	
	Boys	Girls	Boys	Girls
Body-mass index	0.82	0.97	0.95	0.97
Waist-to-height ratio	0.89	0.78	0.81	0.66

## Data Availability

The data presented in this study are available upon reasonable request from the corresponding author.
